# Allometry and phylogeny of within-diaspore biomass allocation: A global analysis

**DOI:** 10.1098/rsbl.2025.0328

**Published:** 2025-11-26

**Authors:** Austin R. Cruz, Brian J. Enquist

**Affiliations:** ^1^ Department of Ecology and Evolutionary Biology, The University of Arizona, Tucson, AZ, USA; ^2^ Santa Fe Institute, Santa Fe, NM USA

**Keywords:** plants, seeds, fruits, diaspore, allometry, scaling, phylogenetics

## Abstract

Allometry, the study of size and its effects on biological traits, provides a quantitative and predictive framework for the evolution of plant allocation strategies. While most studies focus on scaling relationships between distinct structures (e.g., leaves, stems, and roots), the allometry within reproductive structures remains underexplored. Here, we assess the global scaling of biomass within diaspores. We take an ecological and evolutionary perspective to examine the interspecific and phylogenetic allometry of within-diaspore (e.g., seeds, fruit) biomass allocation in plants worldwide. We compiled data on seed mass, fruit mass, and seed dispersal mode (biotic versus abiotic) from open-access databases and the literature for 346 species across 97 families to investigate how diaspore biomass is allocated and then consider how this pattern might be influenced by (i) evolutionary history and (ii) seed dispersal mode. Our results reveal that seed mass scales isometrically with fruit mass. This pattern persists after accounting for phylogenetic relatedness and seed dispersal mode despite a notable trend towards differences in allometric coefficients among groups. Our findings suggest that isometric reproductive scaling is a conserved evolutionary pattern shaped by a combination of selective trade-offs, developmental constraints and historical dispersal syndromes. By integrating scaling theory with evolutionary perspectives, our study advances a predictive framework for understanding plant allometry and its role in shaping reproductive strategies across diverse lineages.

## 1. Introduction

Allometry, the study of how biological traits change (scale) with body size, provides a quantitative and predictive framework in plant evolutionary ecology [1–6]. In plants, allometric relationships specifically provide information about how an organ or structure changes in size in relation to the change in size of another organ or structure. Most size-related variation can be characterized by the power function *Y = βM^α^*, where *M* is a measure of the size of the unit of interest (e.g., organismal mass or organ mass), *Y* is the variable or trait of interest, *β* is a proportionality constant (intercept) that may vary across taxa and environments, and *α* is the allometric scaling exponent [7,8]. Notably, many plant traits have been shown to exhibit quarter-power scaling exponents (e.g., *α* = 1/4, 3/4) that differ significantly from the Euclidean geometric expectation that surface area should scale as a 2/3 power (i.e., *α* = 2/3) of volume [9,10]. These allometric scaling laws hold across plant species [11] and result from highly structured and evolutionarily conserved vascular systems that optimize plant allocation strategies [4,10,12–14].

Resource allocation to plant structures entails trade-offs, and these trade-offs can be quantified using allometric theory [2,15–18]. Most studies focus on allometric scaling between different plant structures (e.g., leaf, stem, and root organs) [5,6,19–23]. In contrast, within-structure allometry, particularly in reproductive organs, remains relatively understudied [24–32]. This gap is significant because reproductive allocation has a direct influence on fitness [33,34]. The diaspore is the dispersal unit that contains seeds and often ‘accessory’ tissue (e.g., the pericarp, or fruit). It offers a promising measure of reproductive investment, as it can be partitioned into seed mass, non-seed ‘accessory’ mass, and seed number [28,29,31].

Previous research on within-diaspore biomass allocation has found initial support for isometric scaling (e.g., *α* = 1) between seed and non-seed mass from regional interspecific data [24–29,31]. Two non-mutually exclusive mechanisms might explain such a pattern. First, isometry may reflect a trade-off between mutualistic and antagonistic selective pressures in plant–animal interactions. Fleshy fruiting structures must attract seed dispersers while deterring seed predators, which frequently overlap within and among species [29,35–42]. This dual role may constrain variation [43]. On the one hand, seed-dispersing animals, which inhabit most major biomes and usually exhibit generalized diets [44–50], may increase the need for plants to produce proportionally larger fruiting structures as a strategy to attract dispersers (i.e., selection for a fruit and seed mass scaling of *α* > 1). On the other hand, many seed-dispersing animals are also seed predators [51], which negatively impact or impose conflicts on plant fitness. For example, seed predators often prefer larger seed sizes; however, larger seeds tend to increase seedling survival [38]. This trade-off might then result in selection for a fruit and seed mass scaling of *α* < 1. Consequently, the observed isometric scaling pattern across species may be generated as a compromise of multiple conflicting selective pressures (see also 52, 53).

Second, diaspore isometry may reflect developmental constraints in growing and metabolically maintaining different-sized reproductive tissues. In short, developing fruits must balance the mechanical and energetic demands of pericarp growth with seed maturation. Since fruits must protect seeds while remaining metabolically sustainable, there may be developmental, physical and energetic limits on the disproportionate enlargement of fruit structures. Additionally, the development of seeds and pericarp tissues relies on shared vascular pathways for nutrient and water supply [54]; excessive investment in one component may reduce the development of the other. Thus, maintaining a balanced, isometric scaling relationship might reflect a simple multiplicative relationship between the number of cells in a reproductive structure and the size of the structure [28,55]. Moreover, strong developmental constraints likely interact with stabilizing selection [56] to produce diaspore isometry. Selection can still drive increases or decreases in total investment (reflected in residual allometric variation or *β*), but such changes occur along the isometric axis, preserving the functional integration of diaspores.

Furthermore, these mechanisms, along with evolutionary history and degrees of phylogenetic relatedness [26,29,46,57], may constrain or diversify scaling relationships within diaspores. An open question, then, is whether diaspore biomass partitioning between fruit and seeds should follow isometry across a broader range of botanical diversity. Understanding how these intrinsic and extrinsic drivers interact, and the patterns that emerge, among diverse taxa is increasingly urgent given the rapid shifts in global reproductive dynamics under climate change [58–61].

Here, we present the first global analysis of within-diaspore allometric scaling from a phylogenetic and ecological perspective. Using data from 346 species across 97 families, we assess how reproductive biomass is partitioned between seeds and accessory tissue. We ask how this allocation scales across species and whether it varies with seed dispersal mode (biotic versus abiotic). Specifically, we address three questions:

– Does fruit mass scale predictably with seed mass across species (i.e., interspecific allometry)?
– Is this pattern influenced by shared ancestry (i.e., phylogenetic allometry)?
– Does seed dispersal mode alter these relationships?

Building on the previous arguments in the literature on how selection should shape variation in diaspore biomass allocation and provisioning, and how this provisioning might be influenced by dispersal mode [28], we test four predictions drawn from allometric theory and ecological expectations. First, fruit mass will scale isometrically (*α* ≈ 1) with seed mass across taxa since species assemblages are often diverse and driven biotically by antagonistic and mutualistic selective pressures and abiotically by environmental filtering. Second, the fruit mass of species with a biotic seed dispersal mode will, on average, scale linearly or superlinearly (*α* ≥ 1) with seed mass since investment into an energetically rewarding fruiting structure should be at least proportional to the cost of seed handling [27]. Third, the fruit mass of species with an abiotic seed dispersal mode will, on average, scale sublinearly (*α* < 1) with seed mass, as investment in an energetically rewarding fruiting structure is minimal or non-existent. Fourth, we test the hypothesis that seed dispersal mode influences the evolutionary correlations and rates of change between different components of plant reproductive effort allocation. We hypothesized that biotic seed dispersal mode allowed for evolutionary increases in both seed and fruit mass [62,63]. This hypothesis predicts that the evolutionary correlation between fruit and seed mass for species with biotic seed dispersal would be different from that for species with abiotic seed dispersal.

## 2. Methods

### (a) Data

We curated open-access data on dry fruit and seed mass for 346 plant species from global datasets spanning multiple biogeographic regions. Data were compiled from the TRY database [64], which provided species’ data from the Amazon [65], Ecuador [66,67] and Colombia [68], the BROT 2.0 plant trait database [69] for Mediterranean Basin species, Chen *et al*. [31] for species from China, Lord & Westoby [28]for species from Australia, and from Lee *et al*. [24] for species from England. Only species with both dry fruit mass and dry seed mass data (recorded in milligrams) were used in the analysis. Seed dispersal mode (biotic versus abiotic) was also recorded for each species. Data on the number of seeds per fruit were not consistently nor reliably reported in the original datasets, and therefore were not included in the analysis (see *‘Seed mass and fruit mass data’* in the electronic supplementary material). For complete details on data, see electronic supplementary material.

### (b) Analysis

To explore how fruit mass scaled with seed mass among plant species, we conducted a major axis regression analysis [70]. We evaluated three separate models: all aggregated data, without the effect of seed dispersal mode (model 1: *fruit mass~seed mass*), with group-specific proportionality constants (*β*) but with a common slope (*α*) (model 2: *fruit mass~seed mass+seed dispersal mode*), and with proportionality constants and slopes free to vary (model 3: *fruit mass~seed mass* × *seed dispersal mode*). To account for the possible effect of shared ancestry among species on fruit and seed mass allometric scaling, we conducted a phylogenetic reduced major axis (*pRMA*) regression. We implemented Pagel’s *λ* to test for phylogenetic signal. Finally, we assessed the evolutionary correlation between reproductive traits and seed dispersal mode by testing for variation in the evolutionary rate matrices. All statistical and phylogenetic analyses were performed using R (v. 4.2.1 [71]). For complete details on analyses, packages and functions, see electronic supplementary material.

## 3. Results

Species in the data were taxonomically diverse, representing 97 families and 242 genera. The 10 families with the most species were Fabaceae (*n* = 46), Rosaceae (*n* = 15), Rubiaceae (*n* = 14), Lecythidaceae (*n* = 11), Melastomataceae (*n* = 11), Adoxaceae (*n* = 9), Euphorbiaceae (*n* = 9), Rhamnaceae (*n* = 9), Sapotaceae (*n* = 9), Lauraceae (*n* = 8) (see electronic supplementary material, figure S1 for complete distribution). There was an extensive range in dry seed mass (mean = 970.59 mg, s.e.m. = 533.28 mg, median = 19.13 mg) and dry fruit mass (mean = 5940.75 mg, s.e.m. = 2646.34 mg, median = 56.79 mg) in our data. The smallest dry seed mass was 0.003 mg (*Bagassa guianensis*, Moraceae) and the largest dry seed mass was 183 230 mg (*Licania micrantha,* Chrysobalanaceae). In comparison, the smallest dry fruit mass was 0.04 mg (*Didymopanax morototoni,* Araliaceae), and the largest dry fruit mass was 700 000 mg (*Theobroma bicolor,* Malvaceae). See electronic supplementary material, figure S2, for dry seed and dry fruit mass distribution for all species combined and for each dataset.

**Figure 1.**
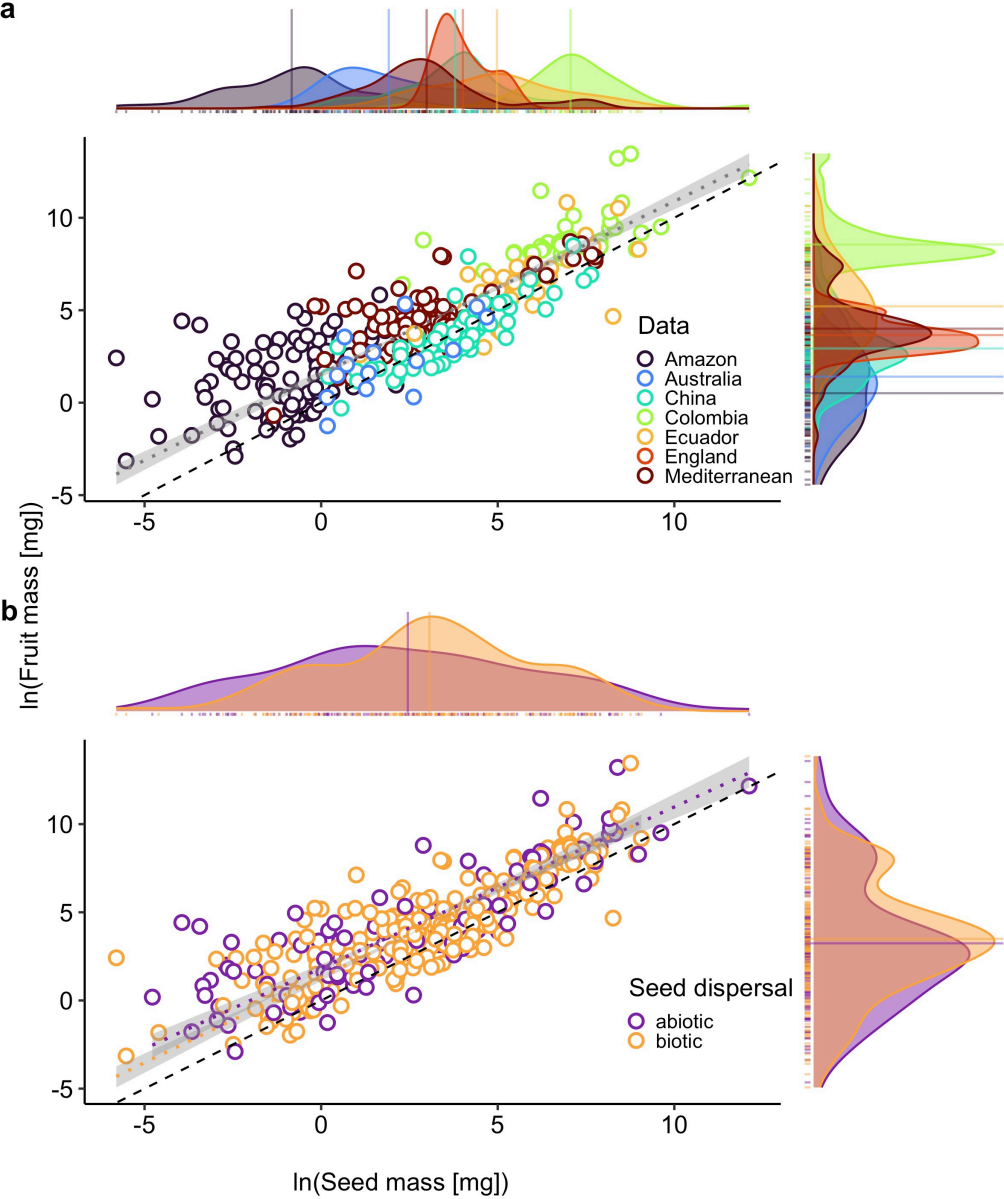
Allometric scaling profiles for all species (a) are grouped by dataset, and (b) are grouped by seed dispersal mode with associated density plots on *x* and *y* axes (group means indicated by solid-coloured lines). Regression lines (dotted) are illustrated with 95% confidence intervals (grey area), and the dashed black line represents 1 : 1 scaling. See the text for statistical details of each panel, and refer to electronic supplementary material, figure S3, for the distributions of data in panel (a)*,* disaggregated by dataset.

Among species, fruit mass scaled isometrically with total seed mass (model 1: *α* = 0.93, 95% CI [0.87, 1.00], *β* = 1.55, 95% CI [1.29, 1.81], *p* = 0.05, *n* = 346; figure 1a, electronic supplementary material, table S1, figure S3). Fruit mass scaled isometrically with seed mass for species with biotic seed dispersal (model 3: *α*_biotic_ = 0.97, 95% CI [0.89, 1.05], *R^2^* = 0.71, *p* = 0.41, *n* = 232; figure 1b), and isometrically for species with abiotic seed dispersal (model 3: *α*_abiotic_= 0.90, 95% CI [0.80, 1.01], *p* = 0.08, *n* = 114; figure 1b). Regression slopes for species with abiotic versus biotic seed dispersal modes were statistically identical (model 3: *χ*²(1) = 0.96, *p* = 0.33), with a common slope indistinguishable from unity (model 3: *χ*²(2) = 3.74, *p* = 0.15), and proportionality constants were not significantly different among groups (model 2: Wald test, *W*(1) = 2.99, *p* = 0.08; *β*_abiotic_ = 1.76, 95% CI [1.38, 2.14], *β*_biotic_ =1.41, 95% CI [1.13, 1.69]). A summary of the regression results is provided in the electronic supplementary material, table S1.

**Figure 2.**
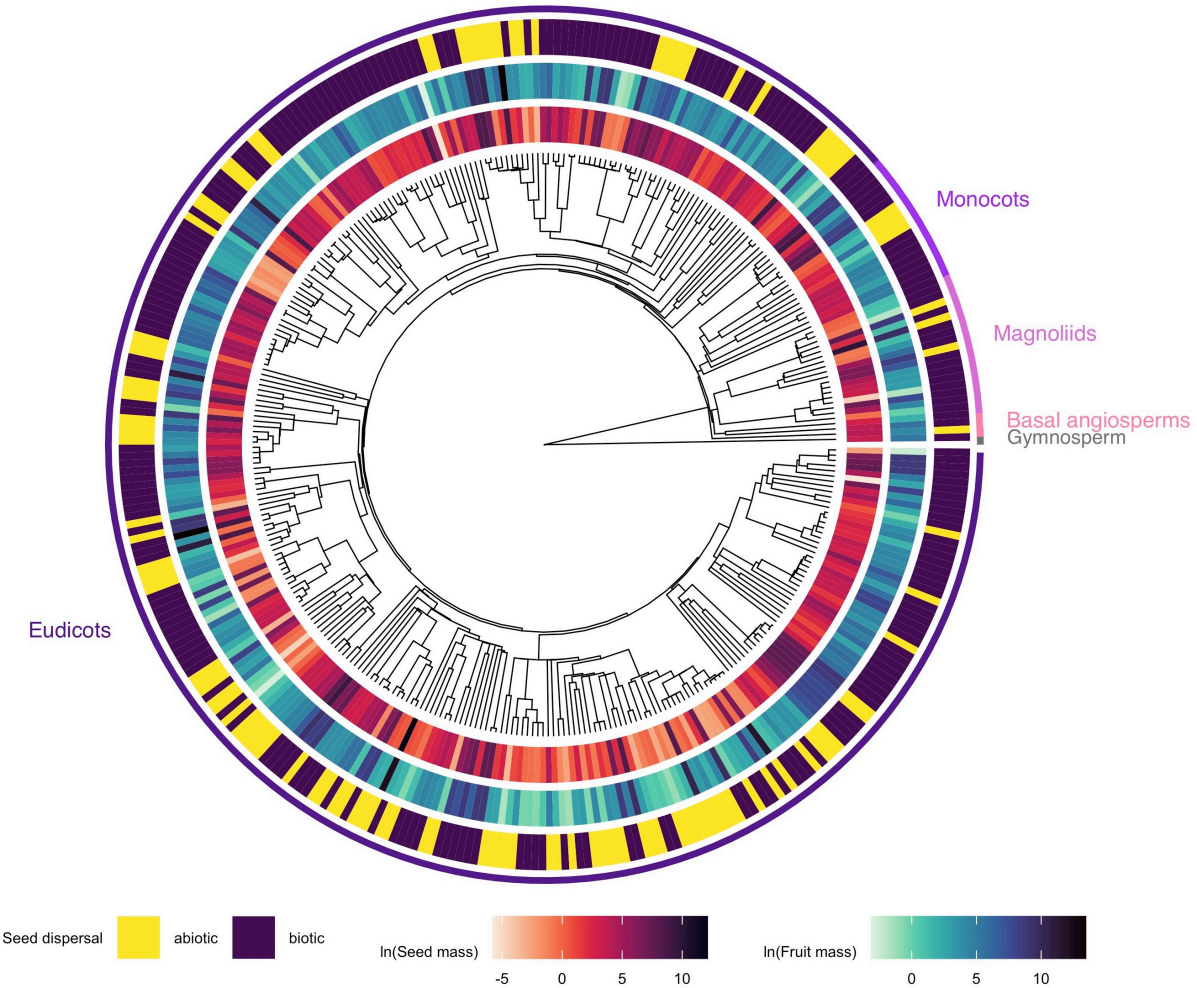
Phylogenetic distribution of plant traits: seed mass (inner ring), fruit mass (middle ring), and seed dispersal mode (outer ring) for all species in the data (*n* = 346) with corresponding legends (left to right: seed dispersal mode (binary), seed mass (natural log transformed), fruit mass (natural log transformed). Outer labels denote major plant taxonomic groups. Phylogenetic backbone was produced from Jin & Qian [72].

At an evolutionary scale, fruit mass and seed mass scaled isometrically when including phylogeny (model 1: *α_pRMA_ =* 1.06, 95% CI [0.61, 1.51], *λ* = 0.60; *β_pRMA_* = −1.44, 95% CI [−1.87, −1.02]; electronic supplementary material, figure S4, table S1). Both seed mass and fruit mass had moderate phylogenetic signals that were significantly different from *λ* = 0 (seed mass: *λ* = 0.36, LR = 16.07, *p* < 0.001; fruit mass: *λ* = 0.40, LR = 12.98, *p* < 0.001; electronic supplementary material, figure S5). The phylogenetic distributions of fruit mass, seed mass and seed dispersal mode are provided in figure 2. Finally, we did not find significant support for a model of variation in the evolutionary rates and correlations between our traits and seed dispersal mode. Specifically, our data were fit better by a model with a single among-trait variance–covariance matrix and a single evolutionary correlation (0.89) between seed mass and fruit mass rather than a multi-matrix model (*χ*²(3) = 3.35, *p* = 0.34; electronic supplementary material, table S2).

## 4. Discussion

Despite its central role in fitness, the evolutionary ecology of within-diaspore biomass allocation has remained understudied. Here, we conducted the first global analysis of within-diaspore allometry, examining how fruit mass scales with seed mass across species, whether shared ancestry influences this relationship, and how dispersal mode affects ecological and evolutionary patterns.

In agreement with our first prediction, we found that fruit mass scaled isometrically (*α* ≈ 1) with seed mass across species. This pattern held across a broad taxonomic and geographic scope, extending previous findings based on smaller and more regionally constrained datasets (see electronic supplementary material, table S3, for a summary). Our results confirm that this isometric scaling pattern holds across a broader range of species, supporting its generality across diverse geographic regions.

Our results were partially consistent with our predictions for the interspecific allometry between fruit mass and seed mass, depending on seed dispersal mode. Previous work investigating animal-dispersed seeds has found support for isometric scaling among species [26], as well as superlinear scaling within species [29] and among families [27]. Although abiotically dispersed seeds exhibited a moderate trend towards sublinear scaling, slopes for each seed dispersal group were indistinguishable from unity. This result may be due to the underrepresentation of abiotically dispersed species, an important consideration since most species lack specific morphological adaptations for seed dispersal (see [42]). Another possibility is that isometry may result from trade-offs among fitness components for abiotically dispersed seeds, specifically the trade-off between seedling establishment and dispersal efficiency [73]. If, on average, selection balances these forces, the result may be a stabilizing pattern of proportional investment.

Notably, a trend towards differences in proportionality constants was observed among the seed dispersal groups. This trend reflects the absolute differences in investment that each group makes in fruit mass. Surprisingly, our results indicated that species in our data with abiotically dispersed seeds invested more on an absolute basis into non-seed ‘accessory’ mass than species with biotically dispersed seeds. One possible explanation for this difference is the megabiota hypothesis [74]. For example, 63% (*n* = 72) of species with abiotically dispersed seeds in our data are from the South American tropics (e.g., Amazon, Colombia and Ecuador), where dry, woody fruiting structures are common. These fruits, often with fleshy endocarp protected by a hard pericarp, are typical of species in Fabaceae [75], the family with the most significant number of species (*n* = 46) and abiotically dispersed species (*n* = 28) in our data. Such structures could require greater investment in the pericarp, inflating the total fruit mass. Many of these fruits may reflect the persistence of anachronistic fruit and dispersal traits dispersed by Pleistocene megafauna [74]. We suspect that the large spread of positive residual variation reflects reproductive traits that evolved to attract now-extinct megafaunal dispersers but that are currently acted upon by abiotic forces [30]. Future work should investigate whether these species, particularly those associated with anachronistic traits, exhibit a detectable allometric signature (e.g., changes in absolute investment shifts, *β*, not relative scaling, *α*) compared with species with contemporary dispersal mechanisms (see [30]). Such empirical research has implications for both ecology and macroevolution, as it helps determine whether investment in woody, protective pericarp reflects adaptive investment in alternative dispersal mechanisms or evolutionary inertia.

We also found that isometric scaling has been evolutionarily conserved among species, indicating that the phylogenetic allometry of fruit mass increased at a rate equal to that at which seed mass increased. Interestingly, the allometric scaling of seed and fruit mass is equivalent in our interspecific and phylogenetically informed models. Such regularity suggests evolutionary canalization [76], where reproductive trait variation is constrained along a predictable axis despite ecological and taxonomic diversity. However, the extent to which different ecological and evolutionary processes contribute to this pattern remains unclear. One possibility is that extant seed dispersers may influence this pattern by interacting with adapted or exapted [77] plant traits, producing functional effects on fitness [30] and community structure [78,79]. Conversely, some extant dispersers may have minimal fitness impacts on plant species exhibiting anachronistic traits [30,74,80].

Our results show that incorporating phylogenetic information significantly reduces the proportionality constant (*β_pRMA_* < *β*). This result indicates that while the relative scaling (*α*) is conserved, the baseline investment (*β*) in accessory (non-seed) tissue for a given seed mass is lineage-specific. These differences may reflect: (i) divergent life-history strategies (e.g., fast- versus slow-growing species), (ii) structural adaptations (e.g., woody pericarps, anachronistic fruits), and/or (iii) biogeographic filters or evolutionary histories of dispersal mode. This finding aligns with the broader prediction from Metabolic Scaling Theory that macroevolution operates within a geometric and physiological scaffolding that channels trait variation along limited, predictable trajectories [2]. This lineage-specific absolute investment is also linked through ‘Corner’s rules’ [81], which describe the architectural and developmental relationships between plant stems and the structures they support. Empirical studies have shown that these rules also apply to reproductive traits, including the scaling of leaf area or size with flower or fruit size (reviewed in [82]). Notably, phylogenetic analyses of reproductive ecology in conifers have demonstrated that dispersal pressures can decouple vegetative and reproductive scaling relationships, specifically the scaling of seed versus pollen. However, strong integration between vegetative and reproductive structures persists [83]. Further research is needed within a phylogenetic framework that examines the extent to which Corner’s rules predict the scaling between vegetative structures and reproductive traits (e.g., seed mass, fruit mass) in angiosperm species associated with different seed dispersal modes.

Despite moderate levels of phylogenetic signal in seed mass and fruit mass [84], these traits were similarly positively correlated, independent of seed dispersal mode, and are best described by a single-rate evolutionary model. We note again that abiotically dispersed species were unevenly represented in the data relative to biotically dispersed species, potentially underpowering the testing of our prediction of the evolutionary influence of seed dispersal mode on our traits. Moving forward, resolving species’ dispersal modes into finer categories beyond a binary classification may provide more dynamic insights into scaling. Other important limitations to note include the taxonomic sampling in our data (e.g., underrepresentation of monocots, a single gymnosperm species) and the use of a phylogenetic backbone (see [72]) that places many taxa as polytomies due to a lack of associated molecular data (see [85]). Expanding taxonomic sampling and resolving phylogenetic relationships (e.g., see [86] for a time-calibrated phylogenetic tree of angiosperms) may reduce potential biases and alter our conclusions. Finally, we acknowledge that including the number of seeds per fruit into our models might enhance our understanding of the effects of seed size/number trade-offs on scaling predictions. Future studies should jointly measure dry seed mass, seed number, and total dry mass allocated to reproduction to assess the degree of their allometric covariation and scaling.

Nevertheless, we emphasize that while mass is a central component of reproductive investment, other traits, such as seed size/number trade-offs [87], seed shape and fruit shape [46,57,88], and nutrient composition [46,47,89] also shape reproductive strategies and likely exhibit their own allometric patterns. Further research on these traits, particularly in terms of dispersal mode and phylogenetic patterns, will enhance our understanding of plant reproductive strategies. Current research in allometric scaling is already developing promising hypotheses and predictions for such traits to enhance agricultural plant breeding and crop production [90,91]. Altogether, our work critically highlights the novel and complementary insights that a phylogenetic approach to within-diaspore biomass allocation offers toward a more predictive evolutionary ecology of plant allometric scaling.

## Data Availability

Full data and analysis code are openly available in Zenodo [92]. Supplementary material is available online [93].
